# Herbal Medicine Characterization Perspectives Using Advanced FTIR Sample Techniques – Diffuse Reflectance (DRIFT) and Photoacoustic Spectroscopy (PAS)

**DOI:** 10.3389/fpls.2020.00356

**Published:** 2020-04-17

**Authors:** Agnese Brangule, Renāte Šukele, Dace Bandere

**Affiliations:** ^1^Department of Human Physiology and Biochemistry, Riga Stradiņš, University, Riga, Latvia; ^2^Department of Pharmaceutical Chemistry, Riga Stradiņš, University, Riga, Latvia

**Keywords:** herbal medicines, FTIR DRIFT, FTIR PAS, cluster analysis, herbal differentiation

## Abstract

This study demonstrates the significant potential of the Fourier transform infrared spectroscopy (FTIR) sampling methods: cantilever-enhanced Fourier transform infrared photoacoustic spectroscopy (FTIR PAS) and diffuse reflectance infrared spectroscopy (FTIR DRIFT) in the field of herbal medicines (HM). In the present work we investigated DRIFT and PAS sampling methods because they do not require sample preparation, samples may be opaque or dark, require small amounts, both liquid and solid samples can be measured, and solid samples can be analyzed on a small scale. Experiments conducted prove high sensitivity, reproducibility and capability in combination with an unsupervised multivariate analysis technique to discriminate important characteristics of HM, such as the identification of plant parts, differentiation of samples by types, and determination of the concentration of extractable compounds in HM.

## Introduction

In today’s world, people are increasingly focusing on healthy lifestyles and the use of herbs. They understand that chemical compounds in herbs can not only help to fight specific diseases but can also be preventative, improving overall health (Enina, 2017).

The chemical composition of herbs may vary depending on the species, location of growth, age, harvesting season, drying conditions, and other factors (Heinrich, 2015). Therefore, comprehensive studies of effective analytical methods are required to make quick and reliable quality control at any stage of herbal medicine (HM) production as well as during the storage process, to obtain feedback (Bostijn et al., 2018; Rehrl et al., 2018).

The World Health Organization and European Pharmacopeia provide guidelines for the assessment of the quality of HM (Bunaciu et al., 2011; Kitanov et al., 2015). In previous studies, there were various techniques that were used to obtain a complete overview of a herbal product, for example, chromatography methods (CM) (Yang et al., 2013; Kitanov et al., 2015). However, CM methods have some critical disadvantages: complicated sample preparation procedures and a long analysis time. Moreover, these are sample-destructive methods (Peerapattana et al., 2015).

Another commonly used method is Fourier transform infrared spectroscopy (FTIR). FTIR methods have been widely used since the 1960s and can be used for both qualitative and quantitative analysis (Stuart, 2004; Smith, 2011). In the field of HMs, the FTIR fingerprint spectra have been used since early 1987, and are used less frequently than CM (Zou et al., 2005). Until now, the introduction of FTIR methods was limited by the complexity of spectra and its interpretation (Rohman et al., 2014). On the other hand, FTIR spectroscopy, in conjunction with multidimensional statistical analysis (Chemometrics), offers a wide scope for HM studies (Kadiroğlu et al., 2018; Miaw et al., 2018). Chemometrics is defined as the application of mathematics and statistics to treat chemical data (Gemperline, 2006), and provide a good opportunity for mining more useful chemical information from the original spectral data by using unsupervised [Principal Component Analysis (PCA), Hierarchical Cluster Analysis (HCA)] and supervised classification methods (Rohman et al., 2019).

The major advantages of FTIR methods are the following: methods are sensitive and non-destructive or only slightly damage the sample; they require minimal sample preparation; small sample quantities are necessary for measuring (Smith, 2011). One of the essential features of FTIR is the possibility to simultaneously determine different components in the same sample from a single instrumental measurement (Moros et al., 2010). In previous studies, the FTIR spectroscopy’s sampling methods transmission and the attenuated total reflection (ATR) was most widely used for diffuse reflectance (DRIFT) and cantilever-enhanced photoacoustic spectroscopy (FTIR PAS) (Legner et al., 2018). The significant benefit of ATR is the ability to measure a wide variety of solid and liquid samples without requiring complex preparations because the ATR measurement is independent of sample thickness and requires small amounts of sample material (Kazarian and Chan, 2006; Rodriguez-Saona and Allendorf, 2011). However, there are significant disadvantages of the ATR sampling method:

• dependence of intensities on a wavenumber and shifts has essential implications on the interpretation of spectra; a particular “ATR correction” function must be used to reduce these differences (Grdadolnik, 2002);
• the spectral absorption area for the ATR sampling method is narrower than other FTIR methods – it starts from 500 to 600 cm^–1^, and important fingerprint region information might be lost.

In the present work, we used DRIFT and PAS sampling methods because they, like ATR, do not require sample preparation, samples may be opaque or dark, the methods require small amounts of sample, both liquid and solid samples can be measured, and solid samples can be analyzed on a small scale. Furthermore, PAS is a little-explored technique for plants (Dias et al., 2018). PAS is based on the photoacoustic effect. The sample is placed in the photoacoustic measurement cell and irradiated with modulated infrared light through a window that is absorbed by the sample at characteristic wavelengths. The heating of the sample generates a pressure wave whose amplitude is detected with a microphone (Gasera, 2010). Photoacoustic spectroscopy is an advantageous method for the measurement and analysis of solid or semi-solid samples, also for powders, fibers, and samples of a very small size, the photoacoustic signal contains information of the surface and inner layers of samples. The shape of the photoacoustic spectrum is independent of the morphology of the sample (Kauppinen et al., 2004; Kuusela and Kauppinen, 2007; Uotila and Kauppinen, 2008).

The purpose of this work is to evaluate the use of FTIR DRIFT with a diamond sampling stick and cantilever-enhanced FTIR PAS in the characterization of HMs.

## Materials and Equipment

### FTIR Sampling Techniques

Cantilever-enhanced photoacoustic spectroscopy (PAS) and diffuse reflectance (DRIFT) spectra were taken with PerkinElmer Spectrum One (450–4000 cm^–1^, resolution of 4 cm^–1^, 10 scans, aperture 8.94 mm, scan speed: 0.2 cm/s). PAS spectra were taken with Gasera PA301 Photoacoustic FTIR accessory (450–4000 cm^–1^, resolution of 8 cm^–1^, 10 scans, aperture 8.94 mm, scan speed: 0.2 cm/s), with the cell being filled with helium gas (flow 0.5 l/min) and a carbon black reference. A unique preparation method was not required for solid, powdered herbals; 0.030–0.040 g of powder was placed in the PAS cell. Each sample was sampled 5 times to reduce the influence of inhomogeneity in the test results.

A modified DRIFT sampling technique was used. Solid, powdered samples were measured directly on the diamond sampling stick. Liquid extracts (60 μl) were placed in the aluminum sampling-cup and evaporated. The raw diffuse reflectance spectra DRIFT will appear different from its transmission equivalent (stronger than expected absorption from weak IR bands). DRIFT spectra were taken in Kubelka-Munk units to compensate for these differences (Greene et al., 2004).

### Plant Material

#### Characterization of Plant Material

Seven medicinal plant species in the form of dried tea samples were measured: chamomile [*Matricaria recutita, 8 different commercial available tea samples* (code – A)], silver birch [*Betula pendula Roth* (B)], hibiscus [*Hibiscus sabdariffa* (C)], peppermint *[Mentha piperita* (D)], cornflower *[Centaurea cyanus* (E)], meadowsweet [*Filipendula ulmaria* (Ms), tansy (*Tanacetum vulgare* (Ta)] (information about samples and measurement conditions in Table 1).

**TABLE 1:** Used materials and methods.

Herbal material	Code in text and figures	Samples	Measurement methods	Measurement conditions
Chamomile (*Matricaria recutita)*	A	8 different commercial available HM samples, Dried powder, extracts (6 pcs.)	DRIFT FTIR PAS FTIR (10 scans, 5 independent sampling for each sample)	450–4000 cm^–1^ DRIFT – 10 scans, resolution 4 cm^–1^ PAS – 10 scans, resolution 8 cm^–1^
Silver birch (*Betula pendula Roth)*	B	1 commercial available HM sample, Dried powder	DRIFT FTIR PAS FTIR (10 scans, 5 independent sampling for each sample)	450–4000 cm^–1^ DRIFT – 10 scans, resolution 4 cm^–1^ PAS – 10 scans, resolution 8 cm^–1^
Hibiscus (*Hibiscus sabdariffa*	C	1 commercial available HM sample, Dried powder	DRIFT FTIR PAS FTIR (10 scans, 5 independent sampling for each sample)	450–4000 cm^–1^ DRIFT – 10 scans, resolution 4 cm^–1^ PAS – 10 scans, resolution 8 cm^–1^
Peppermint *(Mentha piperita)*	D	1 commercial available HM sample, Dried powder	DRIFT FTIR PAS FTIR (10 scans, 5 independent sampling for each sample)	450–4000 cm^–1^ DRIFT – 10 scans, resolution 4 cm^–1^ PAS – 10 scans, resolution 8 cm^–1^
Cornflower *(Centaurea cyanus*	E	1 commercial available HM sample, Dried powder	DRIFT FTIR PAS FTIR (10 scans, 5 independent sampling for each sample)	450–4000 cm^–1^ DRIFT – 10 scans, resolution 4 cm^–1^ PAS – 10 scans, resolution 8 cm^–1^
Meadowsweet (*Filipendula ulmaria)*	Ms	13 different self-collected samples (5 leaves, 8 flowers), Dried powder	DRIFT FTIR (10 scans, 1 independent sampling for each sample)	450–4000 cm^–1^ DRIFT – 10 scans, resolution 4 cm^–1^
Tansy (*Tanacetum vulgare*)	Ta	2 samples (leaves and flowers), Dried powder, Extracts (13 samples)	DRIFT FTIR (10 scans, 1 independent sampling for each sample)	450–4000 cm^–1^ DRIFT – 10 scans, resolution 4 cm^–1^

The herbs meadowsweet (Ms) and tansy (Ta) *(Filipendula ulmaria, Achillea millefolium, Tanacetum vulgare)* were collected while they were blossoming in meadows in Latvia (Sigulda and Ropaži district). The other herb samples were made from herbals produced by Latvian herbal companies.

#### Preparation of Plant Material

All dried herbs were grinded to powder and sifted through a 2 mm sieve. Powders were stored at room temperature for further analysis.

Two extract preparation methods were used:

Method No. 1. The modified extraction method was developed based on literature studies on tannin and phenolic compound extraction methods (Izam, 2012; Nelce et al., 2013). 10 g of dried plant powder was extracted in 100 ml of 30%, 50% or 70% ethanol or acetone in an orbital shaker (180 rpm) for 120 min. at room temperature. The extracts obtained were filtered using Whatman No. 1 filter paper and evaporated to dryness by using a rotary vacuum evaporator to viscose constancy liquid at 60°C and stored at −4°C in an airtight container for further analysis.
Method No. 2. The extraction method was developed based on literature studies on compound extraction for FTIR sampling methods. 5 g of dried plant powder was extracted in 50 ml of ethanol in an occasional shaker for 24 h at room temperature. The extract was filtered using Whatman No. 1 filter paper, and the supernatant was collected and stored at −4°C in an airtight container for further analysis. The residue was collected, dried at room temperature, and stored at room temperature for further analysis (Wulandari et al., 2016).

## Methods

### Spectral Pre-processing

The FTIR spectra were investigated, smoothed, and had their baseline correction and normalization performed with the academic freeware software *SpectraGryph 1.2.14.* The spectra were normalized to the most intense band in the fingerprint region 850–1850 cm^–1^.

### Chemometrics

The PCA and HCA were performed using *SIMCA 14* software. The discrimination was performed in the fingerprint spectral region 850–1850 cm^–1^.

All spectra were smoothed and denoised by a Savitzky – Golay filter (polynomial order 5 and points 15) and the second derivative of the samples was recorded. PCA was used to identify the dominant clusters in the data set (Davis and Mauer, 2010). For the hierarchical cluster analysis, Ward’s algorithm was used (Murtagh and Legendre, 2014). We performed an unsupervised multivariate analysis technique because this method does not require a dependent variable for modeling, it searches for patterns among the independent variables, and groups of samples are formed based on the structure of the variables (Anzanello et al., 2014).

## Results

**FIGURE 1:**
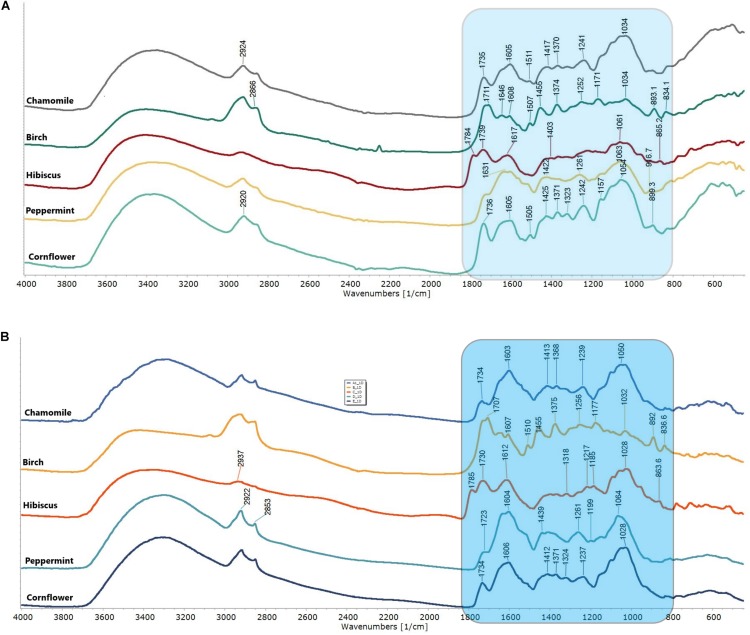
FTIR PAS spectra **(A)** and FTIR DRIFT spectra **(B)** of herbal medicines: chamomile (*Matricaria recutita*), silver birch (*Betula pendula Roth*), hibiscus (*Hibiscus sabdariffa*), peppermint (*Mentha piperita*) *and* cornflower (*Centaurea cyanus*). The fingerprint region 800–850 cm^–1^ is colored in blue.

Figure 1 shows the characteristic FTIR PAS and DRIFT spectra of 5 different herbals: chamomile (A), silver birch (B), hibiscus (C), peppermint (D) and cornflower (E). The FTIR spectra of analyzed HMs occur in 2 spectral regions, showing organic matter and bonds in the sample (850–1850 cm^–1^ and 2700–3200 cm^–1^).

The recorded spectral result shows distinctive spectral patterns in fingerprint region (850−1850 cm^–1^), well defined water absorption band (-O-H stretch, 3200–3400 cm^–1^), well defined, but not specific C-H related peaks (2924 and 2853 cm^–1^ –CH- and –CH_2_-CH_3_). While birch shows a more pronounced spectral pattern with well-defined lines. Chamomile and cornflower show high similarity and predictable lower distinction by PCA and HCA. Specific spectral lines have been identified and summarized in Table 2.

**TABLE 2:** PAS and DRIFT FTIR main absorption bands for fingerprint region of Chamomile **(A)**, Cornflower **(E)**, Birch buds **(B)**, Hibiscus **(C)**, Peppermint **(D)** and assignments.

Sample	DRIFT, cm^–1^	PAS, cm^–1^	Reference, cm^–1^	Bond, stretching	Functional group/compounds	References
Chamomile (A), Cornflower (E)	1734	1735	1737	C = O bond	Luteolin	Zou et al., 2005
	1603	1605	1628	C = O bond	Luteolin	Parlinska-Wojtan et al., 2016
	1374	1370	1375	C–N stretch	Aromatic amines I, II	Dehelean et al., 2007
	1239	1241	1250	C–H stretch and O–H deform. of carboxyl groups and bending of N–H bond	Amide III	Choong et al., 2016
	1102	1105	1109	–C–O	Terpenoids, flavones	Lehto et al., 2018
	1050	1052	1053	C–O–C stretch	Aromatic ethers and polysaccharides	Szymczycha-Madeja et al., 2013
Birch buds (B)	1730	1731	1735 1726–1730	C = O stretching C = O vibration	in unconjugated ketone, carbonyl, and aliphatic groups (xylan); esters, ketones, and aldehydes in hemicelluloses	
	1707	1711	1704 1655–1658	absorbed OH and conjugated CO, HOH, OH bending	in lignin or cellulose absorbed water	
	1455	1455	1453	CH_2_ deformation stretching; CH deformation asymmetric in plane; HCH and OCH in plane bending vibration.	lignin and xylan, lignin and hemicelluloses,	
	1375	1374	1372 1368–1369	aliphatic CH stretching; CH deformation.	in methyl and phenol in cellulose and hemicelluloses, in plane CH bending;	
	1256	1252	1254 1261–1263	stretching of OCO; CO stretching; CO linkages.	guaiacyl ring; in lignin; in guaiacyl aromatic methoxyl groups	
	892	894	895	C-H COC, CCO, and CCH deformation stretching	out of plane glucose ring in cellulose and hemicelluloses and for guaiacyl rings in lignin	
Hibiscus (C)	1785	1784	1788	R_1_R_2_C = CH_2_	Methylene, overtone of δ‘ CH (out of plane)	
	1730	1739	1743	vC = O	Ketone	
	1612	1617	1629	Ortho-CO-C_6_H_4_-OH	Influenced by I,M, and steric effects of substituent	
	1095	1097	1098	-C-H	In-plane CH pending modes	
	1063	1061	1065	-C-H	In-plane CH pending modes	
	1028	1030	1033	vs SO_3_		
Peppermint (D)	1723	1725	1725	C = O vibration	bonded conjugated ketones, aldehydes, quinines and esters	
	1630	1628	1628	C = C vibration of aromatic structures, C = O stretching	amide I and carboxylic acids	
	1439	1422	1441	O-H in plane bend, C-O stretch vibration	carboxylic acids carbonates	
	1261	1261	1250	C–H stretch and O–H deform. of carboxyl groups and bending of N–H bond	Amide III	
	1149	1150	1153	C–O–C stretch	Aromatic ethers and polysaccharides	

Generally, PAS spectra show higher “spectral noises,” specifically in the 1950–2500 cm^–1^ area. However, these “noises” did not affect the FTIR spectra differentiation because they were located outside the fingerprint area and outside the analytically significant area of functional groups. PAS and DRIFT spectra not only show significant differences in spectral line intensities, but also show a similar spectral pattern, and can be directly compared with each other.

### Validation and Repeatability of PAS and DRIFT

To validate the repeatability of the two FTIR sampling methods PAS and DRIFT in the field of HM, firstly, five PAS and DRIFT spectra of each sample were recorded for five very different herbals under the same measurement conditions:

• herbal with flowers native to and grown in Latvia:
  - chamomile (*Matricaria recutita)*, 8 commercial tea samples;
  - cornflower (*Centaurea cyanus)*, 1 sample;

• herbal with buds native to and grown in Latvia - silver birch (*Betula pendula Roth*, B), 1 sample;
• herbal non-native to Latvia - hibiscus (*Hibiscus sabdariff*), 1 sample;
• herbal with peppermint leaves (*Mentha piperita*), 1 sample.

The FTIR spectra were taken in the wavenumber range 400–4000 cm^–1^ (Figure 1). The validation was performed in the fingerprint region 850–1850 cm^–1^. The Pearson Product Moment Correlation coefficient *r* was applied as a support tool to interpret correlation in the fingerprint region at the 850–1850 cm^–1^ using OriginPro2017 software.

The calculated Pearson’s correlations value *r* showed a high correlation for both DRIFT (0.982–0.994) and PAS (0.9992–0.9999) FTIR sampling techniques.

Pearson’s correlation (*r*) was performed for 5 different herbals to obtain correlations between all spectra. The highest Pearson’s *r* values with other herbals showed cornflower (avg 0.745; max 0.936; min 0.473), chamomile (avg 0.742; max 0.936; min 0.534) and peppermint (avg 0.570; max 794; min 0.429). Conversely, the lowest was silver birch (avg 0.505; max 0.584; min 0.429) and hibiscus (avg 0.619; max 0.701; min 0.485). This indicates a greater possibility of differentiation using FTIR sampling techniques, while also proving the high similarity of FTIR fingerprint pattern and highlights the need for a more sophisticated method of discrimination, such as statistical methods of PCA or HCA.

### Differentiation of FTIR PAS and DRIFT Spectra

**FIGURE 2:**
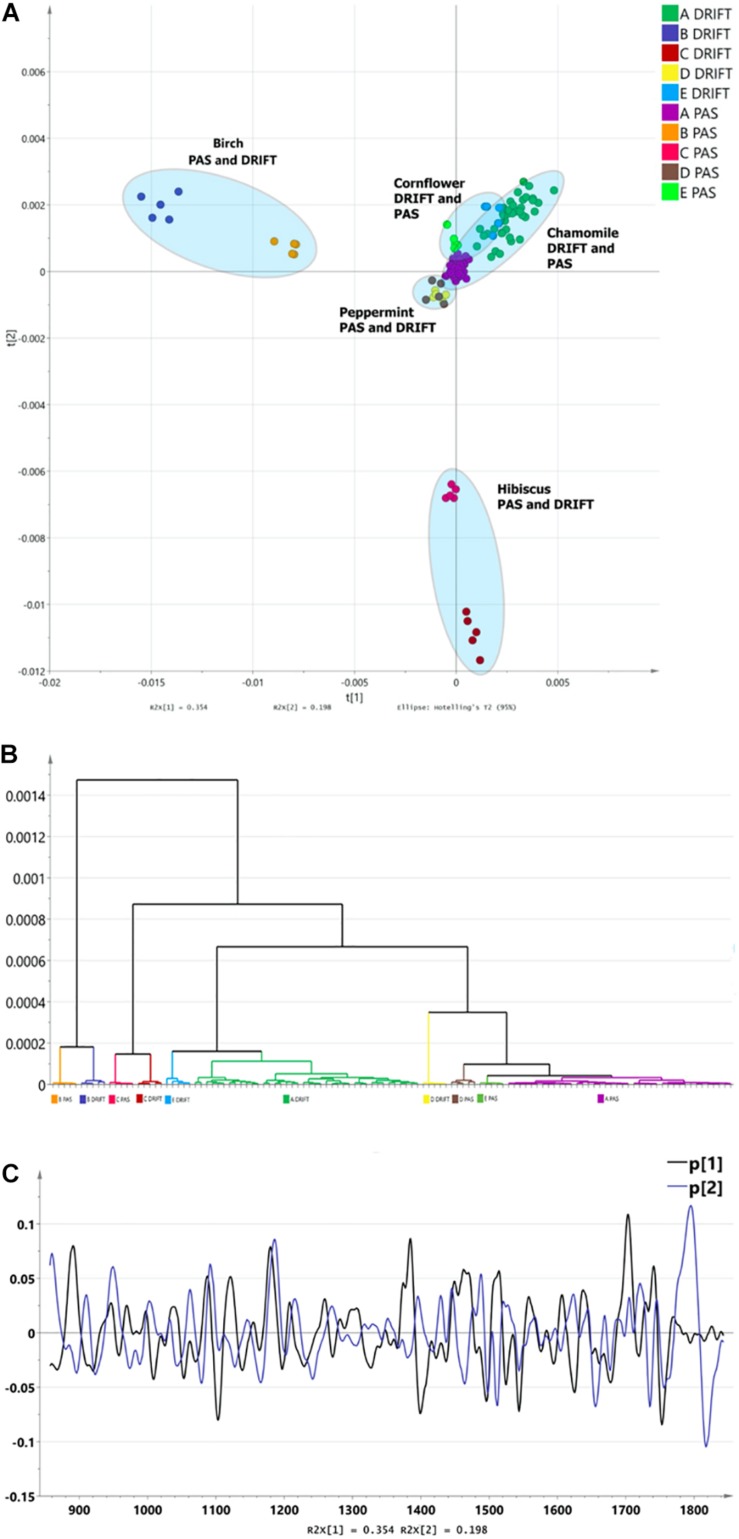
The differentiation of FTIR PAS and DRIFT spectra of herbal medicines: chamomile [*Matricaria recutita*, (code – A)], silver birch [*Betula pendula Roth* (B)], hibiscus [*Hibiscus sabdariffa* (C)], peppermint *[Mentha piperita* (D)], cornflower *[Centaurea cyanus* (E)]. **(A)** The PCA clusters for PAS and DRIFT FTIR sampling methods. **(B)** The HCA dendrogram for PAS and DRIFT FTIR sampling methods. **(C)** Loadings for PAS and DRIFT FTIR sampling methods.

Combining the FTIR methods with unsupervised multidimensional statistical analysis, conclusions about the effect of the sampling method on the obtained results were obtained. To compensate for the differences in differences of the sampling techniques (DRIFT, PAS), second derivatives of fingerprint region spectra were used as input data. Spectral derivatives reduce the impact in differences in spectral sensitivity and peak width. The formation of clusters was depicted in diagrams and dendrograms in Figure 2. Three major clusters in PCA can be identified (Figure 2A). The analysis shows that the greatest influence on cluster formation is not the choice of the FTIR sampling method, but the specificity of HM spectra in the fingerprint region. The HCA shows that differentiation, according to FTIR sampling methods, is possible as well (Figure 2B). PCA1 describes 48.9%, but PCA 9.1%, forming a total of 58% of spectral information. Loadings (Figure 2C) shows a clear difference between PCA1 and PCA2, which provides a clear discrimination for a PCA1/PCA2 diagram and HCA.

**FIGURE 3:**
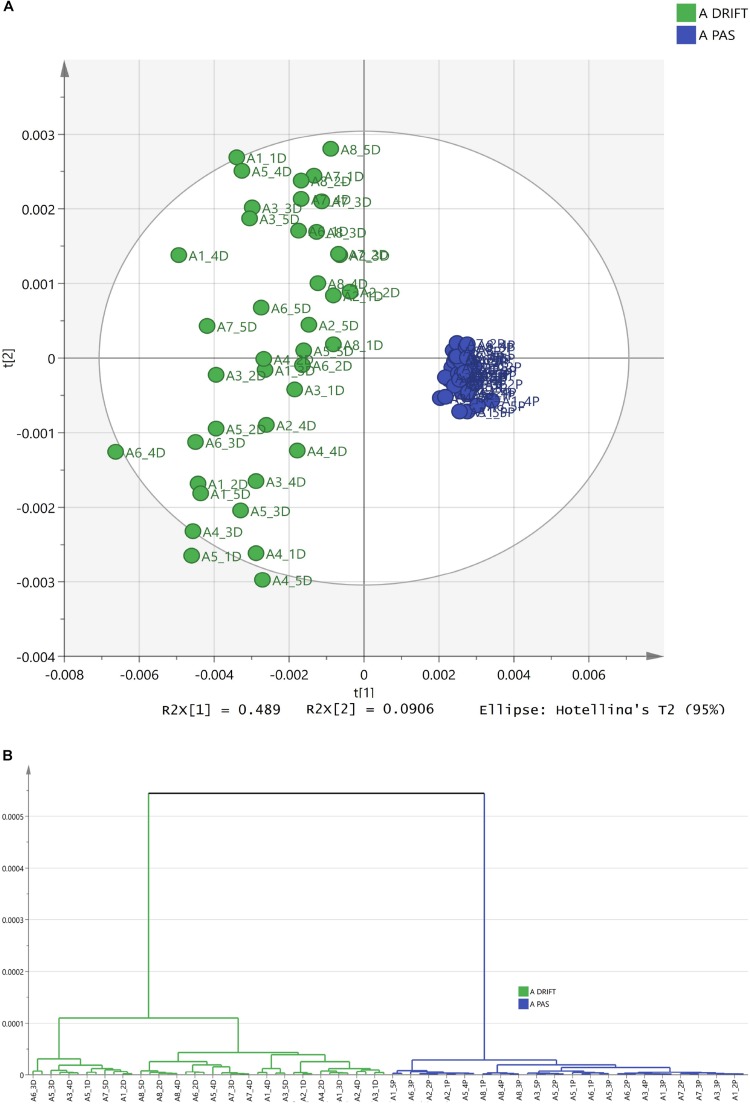
The differentiation of FTIR PAS and DRIFT spectra of chamomile Ch (*Matricaria recutita*). **(A)** The PCA clusters for PAS (blue) and DRIFT (green) FTIR sampling methods. **(B)** The HCA dendrogram for PAS (blue) and DRIFT (green) FTIR sampling methods.

Narrowing the research area, leaving only one HM, for instance, chamomile, Figures 3A,B demonstrate that spectra can be differentiated according to FTIR sampling methods as well.

### Differentiation of HMs Leaves vs. Flowers

The next point of interest was the possibility to differentiate parts of the HM (flowers, leaves, stems). This is a very important factor in the production and quality control of HMs because not always can production regulations specify a proportion between flowers and leaves in dried HM.

**FIGURE 4:**
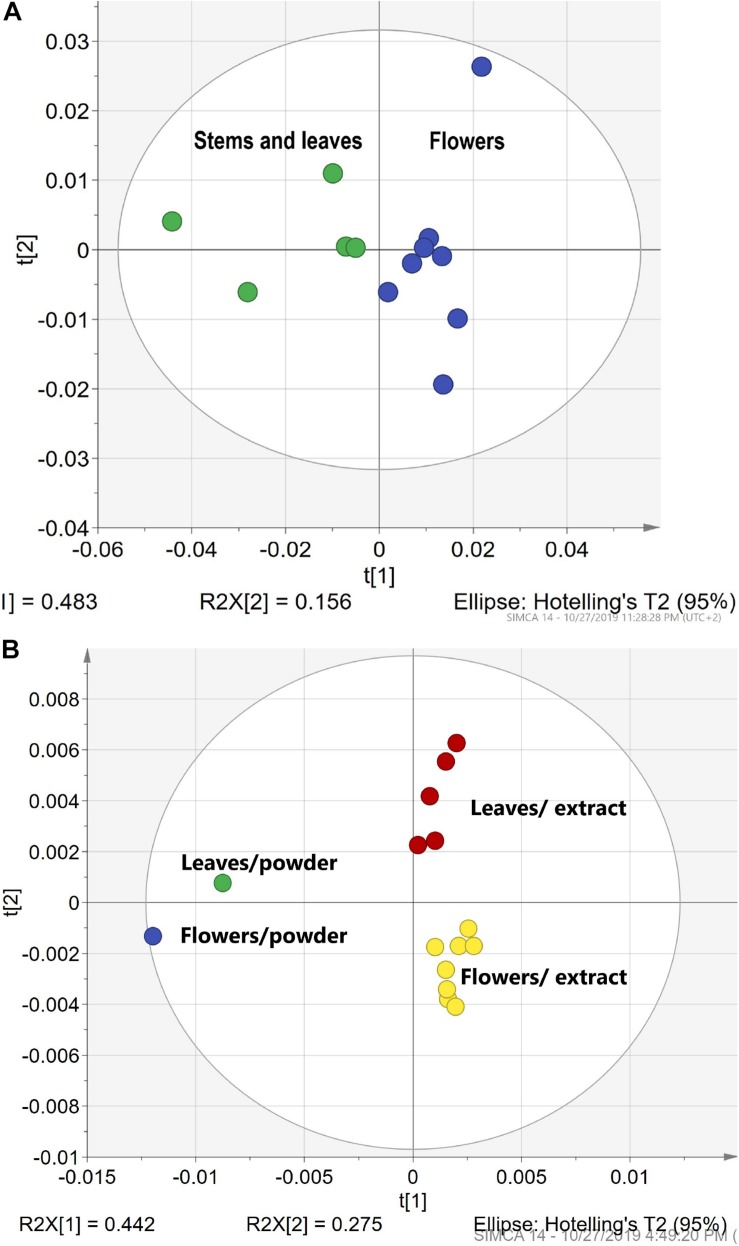
The differentiation of HMs leaves vs. flowers. **(A)** The PCA clusters for meadowsweet Ms *(Filipendula ulmaria)* leaf and flower powders. **(B)** The PCA clusters for tansy Ta *(Tanacetum vulgare)* leaf and flower extracts in ethanol and acetone. Green – leaves and stems; blue- flowers; red – leaf extracts and yellow – flower extracts.

In our experiment, we tested both FTIR spectra of HM leaf and flower powders and HM leaves and flower ethanol and acetone extracts. Figures 4A,B demonstrate separate clusters for leaves and flowers. Figure 4A illustrates a cluster formation for meadowsweet Ms (*Filipendula ulmaria)* leaf and flower powders. Figure 4B shows separate clusters for tansy Ta *(Tanacetum vulgare)* leaf and flower extracts. Moreover, a correlation can be seen between the powder used to make the extract and the leaf or flower extract itself. It demonstrates that the FTIR sampling methods can be used for the differentiation of leaves and flowers in both solid and liquid samples.

### Comparison of Dried Herbals vs. Extracts in Ethanol

**FIGURE 5:**
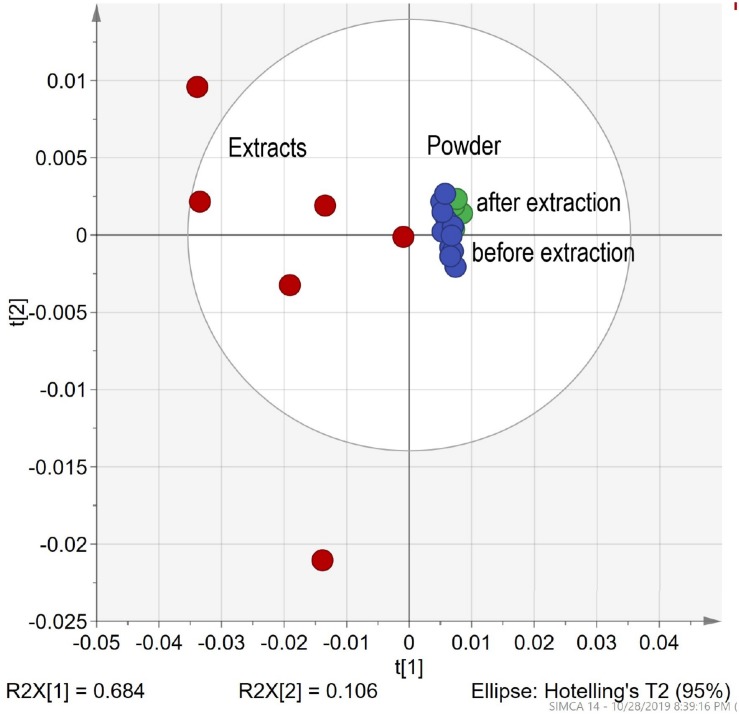
Differentiation HMs powder before extraction vs. extracts in ethanol vs. after extraction. The PCA clusters for chamomile powder before and after extraction and ethanol.

Traditional extracts are made from dried plant material extracted by the appropriate liquid. Method No. 2 was used to prepare 5 extracts of chamomile Ch (*Matricaria recutita*) sourced from different producers. After extraction, the solid residual was collected and dried. All samples (5 powders before extraction, 5 evaporated extracts, and 5 dried residual powders after extraction) measured with the DRIFT method. The fingerprint pattern for powders shows a great deal of similarities. FTIR spectra for extract shows sharper peaks and higher intensity. For evaluation of FTIR spectra, PCA statistical analyses were applied. The resulting PCA1 (71%)/PCA2 (13%) scatter plot (Figure 5) shows characteristics of clusterification according to the sample type. Dispersion across the PCA1 axis could be described by the concentration of the mobile phase in samples. Reducing the concentration in the residual powder after extraction shows a slight shift to the right, and a strong shift in the opposite direction for extracts. The shift intensity correspondent was predicted to be stronger for samples with a higher content of flowers.

## Discussion

A comparison between spectra recorded by PAS and DRIFT sampling methods showed high sensitivity and good discrimination of herbal species based on spectral information. The high complexity of chemical composition and similarity of the main structure of herbal materials adds the complexity of FTIR the spectra interpretation. Traditional methods of spectral interpretation, spectral library search, and line position identification, give very limited information or do not have any practical functionality. The direct comparison of spectral patterns indicates reproducible spectral fingerprints, which could be used for a more sophisticated statistical examination by PCA and HCA methods. The results obtained provide information about the spectral behavior of homogenized herbal and herbal extracts and can be used for establishing identification and discrimination criteria. It has been demonstrated that PAS and DRIFT, in chemometrics, can be a useful experimental tool for the characterization and discrimination of herbals. Also, it must be mentioned that there is a high reproducibility of the PAS FTIR method, but it also demands a high-cost sampling cell and a more advanced FTIR spectrometer with a high-intensity beam and secondary detector connection. DRIFT method gives lower reproducibility, while proving to be more versatile and could be used for powder and liquid samples, while not demanding any specific requirements for the FTIR spectrometer. Despite the high similarity of the fingerprint pattern in the region 850–1850 cm^–1^, the described approach gives promising results for the identification, discrimination, and characterization of MH. Unfortunately, the complexity of the FTIR pattern and limited information about FTIR possibilities for HM limits FTIR for wider usage in HM research. Also, the traditional Diamond ATR sampling cell’s low sensitivity after 1400 cm^–1^ adds another obstacle for broader usage, therefore DRIFT, and PAS prove to be a more appropriate sampling method. The results of the usage of the PAS sampling method gives a high hope for future research: no sampling preparation needed, exceptional reproducibility and comparable spectral pattern with other sampling methods. On the other hand, it also has a few drawbacks: higher costs of PAS cell, lower S/N, and spectral resolution. Also, it should be mentioned that the 8 cm^–1^ resolution used for PAS measurements allows for a better signal to noise.

## Conclusion

Research shows a significant potential of the FTIR sampling methods PAS and DRIFT for a fast and sensitive, reproducible, and non-destructive method for the quality control of HM in a form of powder or liquid extracts. The experiments conducted prove a high sensitivity and capabilities of FTIR method in combination with chemometrics to discriminate important characteristics of MH, such as the identification of plant parts, the concentration of extractable compounds in MH, and differentiation of samples by types. PAS sampling gives unmatchable reproducibility (Pearson’s *r* value = 0.999) for different herbal material but has lower sensitivity and higher spectral noise. A higher sample area analyzed by PAS also improve sampling reproducibility reducing the influence of natural sample inhomogeneity observed by the DRIFT method. PAS gives a clear advantage of high clusterification showed by PCA and HCA. The DRIFT method shows higher versatility for analyzing powder and liquid sample, but lower reproducibility for sampling and spectral measurements (Pearson’s *r* value 0.982–0.994). Both methods show high potential for further research. Additionally, the disadvantages of PAS should be mentioned: higher cost of purchases, use of He gas and a more complex sampling routine.

## Data Availability Statement

The datasets generated for this study are available on request to the corresponding author.

## Author Contributions

AB and RŠ conceived and planned the experiments and carried out the experiments. AB performed all FTIR PAS and FTIR DRIFT measurements and chemometrics. RŠ was responsible for preparing herbal samples and preparing extracts. DB helped by supervising the project and provided critical feedback.

## Conflict of Interest

The authors declare that the research was conducted in the absence of any commercial or financial relationships that could be construed as a potential conflict of interest.

## References

[B1] Anzanello M. J. Ortiz R. S. Limberger R. Mariotti K. (2014). Performance of some supervised and unsupervised multivariate techniques for grouping authentic and unauthentic viagra and cialis. *Egypt. J. Forensic Sci.* 4 83–89. 10.1016/j.ejfs.2014.03.004

[B2] Bostijn N. Hellings M. Van Der Veen M. Vervaet C. De Beer T. (2018). In-line UV spectroscopy for the quantification of low-dose active ingredients during the manufacturing of pharmaceutical semi-solid and liquid formulations. *Anal. Chim. Acta* 12 54–62. 10.1016/j.aca.2018.02.007 29501092

[B3] Bunaciu A. A. Aboul-Enein H. Y. Fleschin S. (2011). Recent applications of Fourier transform infrared spectrophotometry in herbal medicine analysis. *Appl. Spectrosc. Rev.* 46 251–260. 10.1080/05704928.2011.565532 22367902

[B4] Choong Y.-K. Yousof N. Wasiman M. I. Jamal J. A. Ismail Z. (2016). Determination of effects of sample srocessing on Hibiscus sabdariffa L. using tri-step infrared spectroscopy. *J. Anal. Bioanal. Tech.* 7:335 10.4172/2155-9872.1000335

[B5] Davis R. Mauer L. (2010). Fourier transform infrared (FT-IR) spectroscopy: a rapid tool for detection and analysis of foodborne pathogenic bacteria. *Curr. Res. Technol. Educ. Top.* 2 1582–1594.

[B6] Dehelean C. Pinzaru S. C. Peev C. Soica C. Antal D. S. (2007). Characterization of birch tree leaves, buds and bark dry extracts with antitumor activity. *J. Optoelectron. Adv. M.* 9 783–787.

[B7] Dias R. C. E. Valderrama P. Marco P. H. Scholz M. B. D. S. Edelmann M. J. Yeretzian C. (2018). Data on roasted coffee with specific defects analyzed by infrared-photoacoustic spectroscopy and chemometrics. *Data Brief.* 20 242–249. 10.1016/j.dib.2018.08.013 30140720 PMC6104563

[B8] Enina V. (2017). *Veselība Pie Mājas Sliekšna. 100 Populārākie Ārstniecības Augi Latvijā.* Riga: Zvaigzne ABC.

[B9] Gasera (2010). FTIR–PAS Photoacoustic Fourier transform infrared spectroscopy (FTIR–PAS). Available at: http://www.gasera.fi/technology/

[B10] Gemperline P. J. (2006). *Practical Guide to Chemometrics: Ed. by Paul Gemperline*, 2nd Edn Boca Raton: CRC.

[B11] Grdadolnik J. (2002). ATR-FTIR spectroscopy: its advantages and limitations. *Acta Chim. Slov.* 49 631–642.

[B12] Greene E. F. Tauch S. Webb E. Amarasiriwardena D. (2004). Application of diffuse reflectance infrared fourier transform spectroscopy (DRIFTS) for the identification of potential diagenesis and crystallinity changes in teeth. *Microchem. J.* 76 141–149. 10.1016/j.microc.2003.11.006

[B13] Heinrich M. (2015). Quality and safety of herbal medical products: regulation and the need for quality assurance along the value chains. *Br. J. Clin. Pharmacol.* 80 62–66. 10.1111/bcp.12586 25581270 PMC4500325

[B14] Izam A. B. (2012). *Solid-liquid Extraction of Hydrolysable Tannin (galic acid) from Stem Bark of Jatropha Curcas Using Various Type of Extraction.* [dissertation],: University of Malaysia Pahang, Malaysia.

[B15] Kadiroğlu P. Aydemir L. Y. Akcakaya F. G. (2018). Prediction of functional properties of registered chickpea samples using FT-IR spectroscopy and chemometrics. *LWT* 93 463–469. 10.1016/j.lwt.2018.03.080

[B16] Kauppinen J. Wilcken K. Kauppinen I. Koskinen V. (2004). High sensitivity in gas analysis with photoacoustic detection. *Microchem. J.* 76 151–159. 10.1016/j.microc.2003.11.007

[B17] Kazarian S. G. Chan K. L. (2006). Applications of ATR-FTIR spectroscopic imaging to biomedical samples. *Biochim. Biophys. Acta.* 1758 858–867. 10.1016/j.bbamem.2006.02.011 16566893

[B18] Kitanov G. Karcheva-Bahchevanska D. Lukova P. (2015). Comparative analysis of monographs on herbal drugs and herbal drug preparations included in the European Pharmacopoeia (Ph. Eur. 8). *Annales UMCS Pharmacia* 62 20–27.

[B19] Kuusela T. Kauppinen J. (2007). Photoacoustic gas analysis using interferometric cantilever microphone. *Appl. Spectrosc. Rev.* 42 443–474. 10.1080/00102200701421755

[B20] Legner N. Meinen C. Rauber R. R. (2018). Differentiation of agricultural plant cultivars and proveniences using ftir spectroscopy. *Front. Plant Sci.* 9:748. 10.3389/fpls.2018.00748 29951073 PMC6008560

[B21] Lehto J. Louhelainen J. Kłosinńska T. Drożdżek M. Alén R. (2018). Characterization of alkali-extracted wood by FTIR-ATR. Spectroscopy. *Biomass Convers. Biorefin.* 8 847–855. 10.1007/s13399-018-0327-5

[B22] Miaw C. S. W. Assis C. Silva A. R. C. S. Cunha M. L. Sena M. M. de Souza S. V. C. (2018). Determination of main fruits in adulterated nectars by ATR-FTIR spectroscopy combined with multivariate calibration and variable selection methods. *Food Chem.* 15 272–280. 10.1016/j.foodchem.2018.02.015 29548454

[B23] Moros J. Garrigues S. Guardia M. (2010). Vibrational spectroscopy provides a green tool for multi-component analysis, TRAC-Trend. *Anal. Chem.* 29 578–591. 10.1016/j.trac.2009.12.012

[B24] Murtagh F. Legendre P. (2014). Ward’s hierarchical agglomerative clustering method: which algorithms implement ward’s criterion. *J. Classif.* 31 274–295. 10.1007/s00357-014-9161-z

[B25] Nelce M. M. Mahendradatta M. Laga A. Djide N. (2013). Tannin extract of guava leaves (*Psidium guajava* L) variation with concentration organic solvents. *Int. J. Sci. Eng. Technol.* 2 106–110.

[B26] Parlinska-Wojtan M. Kus-Liskiewicz M. Depciuch J. Sadik O. (2016). Green synthesis and antibacterial effects of aqueous colloidal solutions of silver nanoparticles using camomile terpenoids as a combined reducing and capping agent. *Bioprocess Biosyst. Eng.* 39 1213–1223. 10.1007/s00449-016-1599-1594 27083587 PMC4945692

[B27] Peerapattana J. Otsuka K. Hattori Y. Otsuka M. (2015). Quantitative analysis of α-mangostin in hydrophilic ointment using near-infrared spectroscopy. *Drug Dev. Ind. Pharm.* 41 515–521. 10.3109/03639045.2014.884115 24517571

[B28] Rehrl J. Karttunen A. P. Nicolai N. Hörmann T. Horn M. Korhonen O. (2018). Control of three different continuous pharmaceutical manufacturing processes: use of soft sensors. *Int. J. Pharm.* 16 60–72. 10.1016/j.ijpharm.2018.03.027 29555436

[B29] Rodriguez-Saona L. E. Allendorf M. E. (2011). Use of FTIR for rapid authentication and detection of adulteration of food. *Annu. Rev. Food Sci. Technol.* 2 467–483. 10.1146/annurev-food-022510-133750 22129392

[B30] Rohman A. Nugroho A. Lukitaningsih E. Sudjadi (2014). Application of vibrational spectroscopy in combination with chemometrics techniques for authentication of herbal medicine. *Appl. Spectrosc. Rev.* 49 603–613. 10.1080/05704928.2014.882347

[B31] Rohman A. Windarsih A. Hossain M. A. M. (2019). Application of near- and mid-infrared spectroscopy combined with chemometrics for discrimination and authentication of herbal products: a review. *J. Appl. Pharm. Sci.* 9 137–147. 10.7324/JAPS.2019.90319

[B32] Smith B. C. (2011). *Fundamentals of Fourier Transform Infrared Spectroscopy*, 2nd Edn Boca Raton, FL: CRC Press, 10.1201/b10777

[B33] Stuart B. H. (2004). *Infrared Spectroscopy: Fundamentals and Applications.* Hoboken, NJ: Wiley.

[B34] Szymczycha-Madeja A. Welna M. Zyrnicki W. (2013). Multi-Element analysis, bioavailability and fractionation of herbal tea products. *J. Braz. Chem. Soc.* 24 777–787. 10.5935/0103-5053.20130102

[B35] Uotila J. Kauppinen J. (2008). Fourier transform infrared measurement of solid-, liquid-, and gas-phase samples with a single photoacoustic cell. *Appl. Spectrosc.* 62 655–660. 10.1366/000370208784658048 18559153

[B36] Wulandari L. Retnaningtyas Y. Lukman N. Lukman H. (2016). Analysis of flavonoid in medicinal plant extract using infrared spectroscopy and chemometrics. *J. Anal. Methods Chem.* 2016:4696803. 10.1155/2016/4696803 27529051 PMC4977382

[B37] Yang I.-C. Tsai C.-Y. Hsieh K.-W. Yang C.-W. Ouyang F. Lo Y. M. (2013). Integration of SIMCA and near-infrared spectroscopy for rapid and precise identification of herbal medicines. *J. Food Drug Anal.* 21 268–278. 10.1016/j.jfda.2013.07.008

[B38] Zou H.-B. Yang G.-S. Qin Z.-R. Jiang W.-Q. Du A.-Q. Aboul-Enein H. Y. (2005). Progress in quality control of herbal medicine with IR fingerprint spectra. *Anal. Lett.* 38 1457–1475. 10.1081/AL-200062153

